# Early Enhancement in Contrast-Enhanced Computed Tomography Is an Index of *DUSP9*, *SLPI*, *ALDH1L2*, and *SLC1A1* Expression in Canine Hepatocellular Carcinoma: A Preliminary Study

**DOI:** 10.3390/vetsci12020137

**Published:** 2025-02-07

**Authors:** Toshiyuki Tanaka, Tomoki Motegi, Nanami Sumikawa, Misaki Mori, Shohei Kurokawa, Hideo Akiyoshi

**Affiliations:** 1 Laboratory of Veterinary Advanced Diagnosis and Treatment, School of Veterinary Science, Osaka Metropolitan University, Osaka 5988531, Japan; f21724w@omu.ac.jp (T.T.); s.kurokawa@hs-gac.jp (S.K.); 2 Section of Computational Biomedicine, Department of Medicine, Boston University Chobanian & Avedisian School of Medicine, Boston, MA 02118, USA; a-t.motegi@g.ecc.u-tokyo.ac.jp; 3 Laboratory of Veterinary Surgery, School of Veterinary Science, Osaka Metropolitan University, Osaka 5988531, Japan; sdc01019@st.osakafu-u.ac.jp (N.S.); sdc01041@st.osakafu-u.ac.jp (M.M.)

**Keywords:** dog, HCC, liver, radiogenomics, RNA sequence

## Abstract

Canine hepatocellular carcinoma (HCC) is characterized by distinct computed tomography (CT) findings. HCC exhibits tumor heterogeneity, with different genomic information and histopathological features within the same tumor. In human HCC, genetic alterations affect the prognosis and treatment, and research has begun to assess genetic alterations using minimally invasive and reproducible CT. However, the relationship between CT findings and the genomic information of canine HCC is unknown. Early contrast of HCC indicates increased intratumoral neovascular growth. In this study, we aimed to investigate the relationship between enhancement patterns in the arterial phase of CT imaging and gene expression in canine HCC using RNA sequencing. Based on the CT findings, three of the eight dogs studied were classified as having enhancement HCC and five as having non-enhancement HCC. RNA sequencing was performed using the mRNA extracted from the specimens. Eight differentially expressed genes met the cutoff criteria. Among these, *DUSP9*, *SLPI*, and *ALDH1L2* were the most upregulated genes in enhancement HCC, whereas *SLC1A1* was the most downregulated in non-enhancement HCC. Canine HCC may involve different angiogenesis mechanisms. CT findings can be used to assess the gene expression status in canine HCC and may add new value to CT imaging.

## Simple Summary

Genetic alterations affect the prognosis and treatment of human hepatocellular carcinoma (HCC). Research has begun to assess genetic alterations using minimally invasive and reproducible computed tomography (CT). However, the relationship between CT findings and the genomic information of canine HCC is unknown. In this study, we aimed to investigate the relationship between enhancement patterns in the arterial phase of CT imaging and gene expression in canine HCC using RNA sequencing. Based on the CT findings, three of the eight dogs studied were classified as having enhancement HCC and five as having non-enhancement HCC. RNA sequencing was performed using the mRNA extracted from the specimens. *DUSP9*, *SLPI*, and *ALDH1L2* were the most upregulated genes in enhancement HCC, whereas *SLC1A1* was the most downregulated gene in non-enhancement HCC. Canine HCC may involve different angiogenesis mechanisms. CT findings can be used to assess the gene expression status in canine HCC and may add new value to CT imaging.

## 1. Introduction

Computed tomography (CT) is often used in veterinary medicine to differentiate liver tumors and evaluate their site of origin. Canine hepatocellular carcinoma (HCC) is characterized by distinct CT findings in individual cases, including early, heterogeneous, and reduced enhancement [1,2,3].

CT, positron emission tomography, and magnetic resonance imaging provide a three-dimensional global view of imaging features that reflect tumor heterogeneity [4,5]. HCC exhibits tumor heterogeneity with different types of genomic information and histopathological features within the same tumor (intratumoral heterogeneity) [4]. Genetic alterations in human HCC affect prognosis and treatment outcomes [6]. The diagnosis and treatment of HCC in humans are based on genomic and histopathological features [4]. However, caution should be exercised when assessing genomic information from biopsy tissues because the information obtained is valid only for a portion of the tumor and not for the entire HCC [7]. Imaging studies have been used to evaluate entire tumors in a minimally invasive and reproducible manner [4]. Although limited, CT and magnetic resonance imaging findings have been associated with gene expression profiles in human HCC [8,9]. Grouping various CT findings, such as the internal arteries, has revealed specific genomic information for each finding [8]. However, there have been limited reports on gene expression in canine HCC [10,11,12].

RNA sequencing (RNA-Seq) is an indispensable tool for transcriptome-wide analysis that enables the study of differential gene expression and mRNA splicing [13]. Although the CT findings of canine HCC include early contrast and no contrast-enhancing effects, the molecular differences and cancer characteristics associated with these typical findings remain to be elucidated. Early contrast in HCC indicates increased intratumoral neovascular growth [14,15]. We hypothesized that the molecular differences between early contrast-enhancing and non-contrast-enhancing HCCs would reveal the mechanisms of angiogenesis and characterize HCC in dogs. In this study, we aimed to investigate the relationship between enhancement patterns in the arterial phase of CT imaging and gene expression in canine HCC using RNA-Seq.

## 2. Materials and Methods

For this study, dogs histopathologically diagnosed with HCC (n = 8) and those with normal livers (n = 4) were selected. Liver specimens were obtained during liver tumor resection at Osaka Metropolitan University Veterinary Medical Center, Japan. The inclusion criteria for this study were as follows: (1) histopathological diagnosis of HCC and (2) contrast-enhanced CT examination. The exclusion criterion was the presence of different CT findings for the same HCC. Specimens were collected in conjunction with CT imaging findings to match the exact locations of the enhancement and non-enhancement areas in the arterial phase. Livers of dogs with no histopathological abnormalities were used as normal liver specimens. Specimens for tissue banking were placed in a liquid nitrogen bath, snap-frozen, and stored at −80 °C.

CT was performed using an Activion 16 (Canon Medical Systems Corporation, Tochigi, Japan) multidetector 16-slice CT scanner in helical scan mode, according to a previously reported protocol [16]. All dogs were placed under general anesthesia, ventilated, and in a supine position. Ventilation was paused during image authacquisition to induce apnea. For contrast-enhanced studies, 2 mL/kg iohexol, a non-ionic contrast medium (300 mgI/mL; Ioverin 300; Teva Pharma Japan, Inc., Aichi, Japan), was administered. The injection duration was 20 s. Contrast-enhanced studies were performed during the arterial (20 s after injection of the contrast medium), portal (60 s after injection of the contrast medium), and equilibrium (180 s after injection of the contrast medium) phases. The CT images were displayed in an abdominal window setting (window level = 35 Hounsfield Unit (HU), window width = 360 HU) to assess the liver neoplasms on a computer workstation using digital imaging and communications in medicine image-viewing software (Horos software ver. 2.4.1, Horos Project, Minneapolis, MN, USA). The CT images were reviewed by a veterinarian with over 10 years of experience as a radiologist. The mean attenuation of the HCC and the adjacent liver was measured on arterial-phase post-contrast images. The region of interest was manually drawn thrice to encompass the lesion. The mean HU values and standard deviations (SDs) of the lesions in the arterial-phase images were calculated. Necrotic and cystic areas confirmed both grossly and histopathologically were excluded when defining the CT findings. Statistical calculations were performed using R version 4.4.2 (R Core Team (2024). _R: A Language and Environment for Statistical Computing_. R Foundation for Statistical Computing, Vienna, Austria. <https://www.R-project.org/> accessed on 2 December 2024). Normalization of attenuation was assessed using the Shapiro–Wilk test, which indicated that parametric testing was required. Results with *p*-values less than 0.05 were considered significant. According to the literature [8], the enhancement of HCC is defined as the presence of discrete arteries within the tumor and a statistically higher enhancement of the tumor compared with the adjacent liver. A non-enhancement tumor was defined as one that exhibited statistically lesser enhancement in the arterial phase than in the adjacent liver.

Total RNA was extracted from frozen liver tissues using the NucleoSpin^®^ RNA Plus kit (Takara Bio Inc., Shiga, Japan) following the manufacturer’s protocol. The purity and concentration of the isolated RNA were assessed by measuring the absorbance ratio at 260/280 nm (A260/A280) using an Eppendorf Biophotometer (Eppendorf, Hamburg, Germany).

The isolated samples were analyzed using an Agilent 2100 Bioanalyzer (Agilent Technologies, Santa Clara, CA, USA), and samples with an RNA Integrity Number greater than 7.0 were selected for further experiments. RNA-Seq was performed using a NextSeq 500 system (Illumina K.K., Osaka, Japan). Quality control and adaptor trimming of the obtained fastq files for each sample were performed using FASTP. After trimming, the raw count data were obtained using a previously published pipeline [17]. Differentially expressed genes (DEGs) were identified using a generalized linear model in edgeR-based R packages (TCC v1.38.0), and significant groups were distinguished using the baySeq package (v2.32.0) in R. The *q* values were calculated from the *p*-values using the Benjamini–Hochberg method, and the false discovery rate was set at *q* < 0.01.

## 3. Results

**Figure 1:**
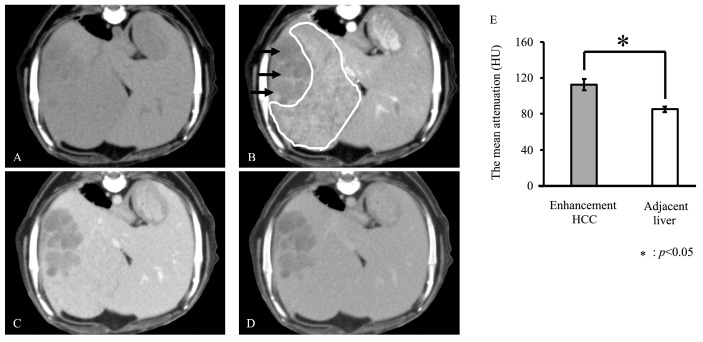
Representative axial phase computed tomography (CT) images of the enhancement hepatocellular carcinoma (HCC) (**A**–**D**) and the mean HU of enhancement HCC and adjacent liver in arterial-phase images (**E**). Images depict pre-contrast (**A**), arterial (**B**), portal (**C**), and equilibrium (**D**) phases. Enhancement HCC was defined by the presence of discrete arteries within the tumor and a statistically higher enhancement in the arterial phase than in the adjacent liver ((**E**), *p* < 0.05). White circle: enhancement HCC; black arrows: necrotic and cystic areas in the HCC.

**Figure 2:**
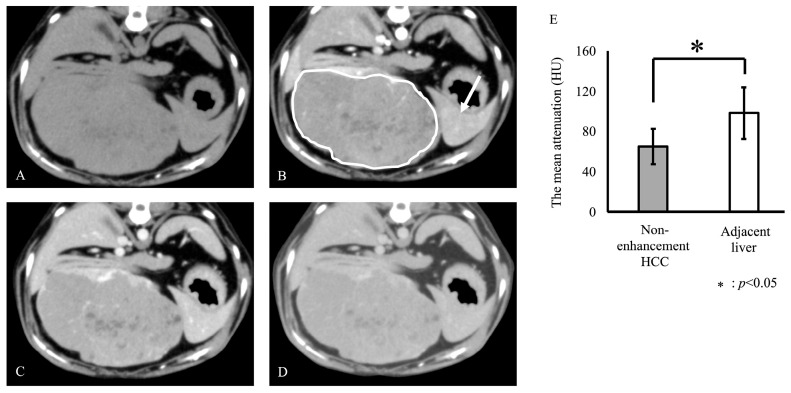
Representative axial phase computed tomography (CT) images of the non-enhancement hepatocellular carcinoma (HCC) (**A**–**D**) and the mean HU of the non-enhancement HCC and adjacent liver in arterial-phase images (**E**). Images depict pre-contrast (**A**), arterial (**B**), portal (**C**), and equilibrium (**D**) phases. Non-enhancement HCC was defined as a statistically lesser enhancement in the arterial phase than that in the adjacent liver ((**E**), *p* < 0.05). White circle: non-enhancement HCC; white arrow: adjacent liver.

Based on the CT findings of HCC, three of the eight dogs were classified as having enhancement HCC and five as having non-enhancement HCC. On enhancement HCC, the mean HU was 112.3 ± 6.4 and 85.0 ± 3.1 for lesion and adjacent liver, respectively. The mean HU of enhancement HCC in arterial-phase images was significantly higher than that of the adjacent liver (*p* < 0.05, Figure 1). On non-enhancement HCC, the mean HU was 65.2 ± 17.4 and 98.3 ± 25.8 for lesion and adjacent liver, respectively. The mean HU of non-enhancement HCC in arterial-phase images was significantly lower than that of the adjacent liver tissue (*p* < 0.05, Figure 2). Representative figures of the enhancement and non-enhancement HCC are shown in Figure 1 and Figure 2.

The dogs with enhancement HCC included one neutered male, one intact male, and one intact female. The mean age of the dogs was 10.8 ± 2.9 year (mean ± SD). The dog breeds were as follows: one West Highland White Terrier, one Toy Poodle, and one Dachshund. Dogs with non-enhancement HCC included one intact male, one neutered female, and three intact females. The mean age of the dogs was 10.4 ± 2.7 years (mean ± SD). The dog breeds included three Shiba Inu, one Border Collie, and one Brussels Griffon. All dogs with HCC underwent surgical removal. The dogs were followed up without treatment for two years. None of the dogs with enhancement or non-enhancement HCC had postoperative recurrence or metastasis. The dogs with normal livers included four intact female Beagles with a mean age of 3.2 ± 1.4 years (mean ± SD). Dogs with normal livers, enhancement HCC, and non-enhancement HCC were grouped as G1, G2, and G3, respectively. The clinical findings are summarized in Table 1. Eight DEGs met the cutoff criteria (Table 2). Among these, *DUSP9*, *SLPI*, and *ALDH1L2* were the most upregulated in enhancement HCC, whereas *TRPV6* was the most upregulated in the normal liver. Furthermore, *SLC1A1* was the most downregulated in non-enhancement HCC, whereas *TOP2A* and *CENPF* were the most downregulated in the normal liver. One gene (Ensembl gene ID: ENSCAFG00000047783) was excluded from the analyses because its status was retired in the ROS_Cfam_1.0 assembly.

## 4. Discussion

In this study, *DUSP9*, *SLPI*, and *ALDH1L2* were upregulated in canine-enhancement HCC. The enhancement of HCC in the arterial phase indicates increased intratumoral neovascular growth [14,15]. In human HCC, *VEGF*, *FGF*, *PDGF*, and *ANGPT* promote angiogenesis [18]. However, in this study, enhancement HCC did not show overexpression of *VEGF*, *FGF*, *PDGF*, or *ANGPT* (Supplementary Material Table S1). Secretory leukocyte protease inhibitors (SLPIs) can affect tumor cell behavior and sinusoidal vasculature formation [19]. SLPI is a promising anti-inflammatory agent that is synthesized and released mainly by epithelial and inflammatory cells [20,21]. However, SLPI-induced angiogenesis is not driven by the usual angiogenic processes (endothelial cell proliferation, vascular extension, and tube formation) but rather occurs through a specific remodeling process in coordination with other molecules such as hypoxia-related angiogenic factors and necrosis-induced cytokines [19]. Considering the expression levels of *VEGF*, *FGF*, *PDGF*, and *ANGPT*, *SLPI* may be primarily involved in angiogenesis in canine-enhancement HCC, unlike in humans. Unlike human HCC, *SLPI* in canine HCC may be involved in angiogenesis. This study only assessed the mRNA expression levels in canine HCC. Further investigation using histopathological techniques is required to clarify the relationship between *SLPI* and angiogenesis.

A strong correlation exists between aberrant SLPI expression and the development of various human cancers, including lung, ovarian, cervical, neck, and pancreatic tumors [21]. In human HCC, SLPI suppressed the proliferation, migration, and invasion capabilities of HCC cells in vitro, whereas ectopic SLPI expression inhibited their tumorigenicity in vivo [22]. SLPI regulates the proliferation, migration, and invasion capabilities of HCC cells via apoptosis through the mitogen-activated protein kinase (MAPK) signaling pathway [22], indicating its potential as both a tumor suppressor and a biomarker for HCC prognosis and treatment [21]. In this study, neither enhancement nor non-enhancement HCC showed postoperative recurrence or metastasis. Further studies in canine HCC cases are required to determine the relationship between *SLPI* expression and HCC prognosis.

Dual-specificity phosphatase (DUSP), also referred to as MAPK phosphatase, modulates MAPK activity by dephosphorylating phosphotyrosine and phosphoserine/phosphothreonine residues on extracellular signal-regulated kinase, c-Jun N-terminal kinase, and p38 [23]. DUSP has been increasingly recognized for participating in various cellular processes [24,25]. The expression of DUSP9 is elevated in human HCC [26,27]. Elevated DUSP9 expression is linked to reduced disease-free survival and an increased risk of recurrence following liver resection [27]. In humans, reports on the expression of DUSP9 in HCC are conflicting, with a few studies reporting decreased expression [28]. Overexpression of DUSP9 correlates with a good prognosis [28].

In humans, *ALDH1L2* mRNA expression is associated with the histopathological grade of HCC [29]. Grades 1–3 of HCC indicate significantly higher aldehyde dehydrogenase 1 family member L2 (ALDH1L2) expression than that in the normal liver, while grade 4 indicates no difference [29]. To the best of our knowledge, no grading scale is currently available for canine HCC [30]. In breast cancer, ALDH1L2 suppresses reactive oxygen species production and is involved in the MAPK pathway [31]. The mechanism of action of ALDH1L2 in human HCC remains unclear; however, in canine HCC, *SLPI*, *DUSP9*, and *ALDH1L2* may be involved in the MAPK pathway. Furthermore, the expression of *SLPI*, *DUSP9*, and *ALDH1L2* in canine HCC and their effects remain unclear. Considering the effects of *SLPI*, *DUSP9*, and *ALDH1L2* in humans, overexpression of these genes in canine-enhancement HCC may indicate lower malignancy. However, a massive canine HCC indicates low systemic progression and a favorable long-term prognosis [32]. Further studies are required to investigate the relationship between the overexpression of *SLPI*, *DUSP9*, and *ALDH1L2* and their influence on the enhancement of HCC.

Solute carrier family 1 (SLC1)A1 functions as a glutamate transporter in neurons, retinal ganglion cells, and glial cells [33,34,35,36]. SLC1A1 is expressed outside the central nervous system, particularly in the intestine, liver, heart, skeletal muscle, kidneys, placenta, sciatic nerve, dorsal root ganglion, and primary afferent fibers terminating in the dorsal spinal horn [33]. Outside the brain, SLC1A1 appears to be the main glutamate and aspartate transporter in several cell types [33]. However, few studies have investigated the relationship between *SLC1A1* expression and cancer development. In human liver cancer, *SLC1A1* is downregulated compared with that in the normal liver [37]. In this study, *SLC1A1* was downregulated in non-enhancement HCC compared with that in normal liver. However, the implications of this downregulation in HCC remain unclear. In human lung adenocarcinoma, low *SLC1A1* expression is correlated with tumor stage, histological subtype, nodal metastasis status, and poor overall survival [37]. Further studies are required to assess the effects of *SLC1A1* downregulation on canine HCC.

CT is a noninvasive method for visualizing the internal tissues of the body, and the resulting images are crucial for clinical decision-making, including diagnosis and treatment [38]. The findings of this study suggest that CT findings can represent differences in mRNA expression levels and may help assess genomic information in the future. CT findings may have the potential to predict the prognosis and treatment response. This study has the potential to increase the amount of information obtained from CT findings and facilitate the adoption of noninvasive genetic evaluation in clinical practice.

This study had certain limitations. First, this study included a biased and small number of dogs with enhancement HCC. Benign masses, such as nodular hyperplasia and hepatocellular adenomas, show significant enhancement compared with the adjacent liver during the arterial phase [1,2,3]. No preoperative biopsy was performed at our institution. Therefore, few cases were followed up without surgery and were not included in this study. This may have resulted in a low number of cases of enhancement HCC. This study evaluated the enhancement in the arterial phase using CT findings and genomic information. However, HCC presents various CT characteristics, such as contrast effects in the portal and equilibrium phases, necrosis, and tumor size. Combining multiple CT findings may allow for a more accurate assessment of genomic information. Moreover, we did not investigate the effect of *DUSP9*, *SLPI*, or *ALDH1L2* on angiogenesis. Only DEGs observed in canine-enhancement HCC were identified, and how *DUSP9*, *SLPI*, and *ALDH1L2* influence the imaging findings remains unclear. Therefore, further studies on canine HCC cases are required to investigate the relationship between CT findings and genomic information. Second, the age differences between the normal liver and hepatocellular carcinoma may have influenced the DEGs in HCC. The main objective of this study was to determine differences in genomic information between enhancement and non-enhancement HCC. Therefore, we hypothesized that age-related genomic changes do not significantly affect healthy dogs. Third, protein expression levels could not be evaluated in this study; although RNA samples were collected, tissue samples were not preserved for protein expression analyses. Therefore, the function and activity of DUSP9, ALDH1L2, and SLPI could not be evaluated.

## 5. Conclusions

CT findings in canine HCC may indicate differences in mRNA expression levels. These findings can predict the gene expression status in canine HCC, thereby adding new value to CT imaging.

## Figures and Tables

**Table 1:** The clinical findings in dogs with HCC and normal livers.

Group	CT Examination	Age	Sex	Bleed	Recurrence	Metastasis
G1	NL	3.3	IF	beagle	N/A	N/A
G1	NL	4.8	IF	beagle	N/A	N/A
G1	NL	1.4	IF	beagle	N/A	N/A
G1	NL	3.3	IF	beagle	N/A	N/A
G2	enhancement	13	IF	Dachshund	-	-
G2	enhancement	12	IM	West Highland White Terrier	-	-
G2	enhancement	7.5	CM	Toy Poodle	-	-
G3	non-enhancement	14.4	IF	Shiba	-	-
G3	non-enhancement	12	IF	Border Collies	-	-
G3	non-enhancement	9	SF	Brussels griffon	-	-
G3	non-enhancement	8	IM	Shiba	-	-
G3	non-enhancement	8.6	IF	Shiba	-	-

NL, Normal liver; CM, cast male; IM, intact male; SF, spayed female; IF, intact female.

**Table 2:** DEGs as q-value < 0.01 among enhancement HCC, non—enhancement HCC, and normal liver.

Ensembl Gene ID	Gene Symbol		Counts per Million Mapped Reads								
			G1					G2			
			NL	NL	NL		NL	E-HCC	E-HCC		E-HCC
ENSCAFG00000019241	DUSP9		0	0	1		0	699	2		139
ENSCAFG00000028626	SLPI		18	2	3		2	269	3006		546
ENSCAFG00000001911	ALDH1L2		9	5	5		9	305	16		1775
ENSCAFG00000047783	N/A		43.7	123.42	60.84		70.61	1.4	0		4.94
ENSCAFG00000016090	TOP2A		16	11	6		18	116	175		620
ENSCAFG00000025465	TRPV6		317	123	191		153	55	1		56
ENSCAFG00000012593	CENPF		4	4	4		4	24	90		280
ENSCAFG00000002067	SLC1A1		1879	1818	1332		2516	682	815		1204
G3								*q*-value		DEG order	
NE-HCC	NE-HCC	NE-HCC		NE-HCC		NE-HCC					
0	0	1		0		0		0.004863		G2 > other	
33	66	28		18		128		0.004863		G2 > other	
22	14	33		18		14		0.005376		G2 > other	
1.56	0	1.44		3.71		2.1		0.005387		0	
254	176	673		398		166		0.008041		other > G1	
0	3	1		0		0		0.009765		G1 > other	
143	131	213		75		59		0.009765		other > G1	
0	4	44		0		0		0.009828		other > G3	

Normal livers were grouped as G1, enhancement HCC as G2, and non-enhancement HCC as G3. NL, normal liver; E-HCC, enhancement HCC; NE-HCC, non-enhancement HCC.

## Data Availability

The data have been deposited with links to BioProject, accession number PRJDB 18013, in the DDBJ BioProject database (https://identifiers.org/bioproject:PRJDB18013) accessd on 2 December 2024. HCC cases with CT images included in PRJDB 18013 were used for the analysis. Normal liver samples included SAMD00771057, SAMD00771058, SAMD00771059, and SAMD00771060. Enhancement HCC samples included SAMD00771063, SAMD00771066, and SAMD00771067. Non-enhancement HCC samples included SAMD00771061, SAMD00771062, SAMD00771064, SAMD00771065, and SAMD00771068.

## Supplementary Materials

The following supporting information can be downloaded at: https://www.mdpi.com/article/10.3390/vetsci12020137/s1. Table S1. All DEGs among enhancement HCC, non-enhancement HCC, and normal liver.

## Abbreviations

CT	Computed tomography
DEG	Differentially expressed gene
DUSPs	Dual-specificity phosphatases
HCC	Hepatocellular carcinoma
MAPK	Mitogen-activated protein kinases
MRI	Magnetic resonance imaging
RNA-Seq	RNA sequencing
SLC1	Solute carrier family 1
SLPIs	Secretory leukocyte protease inhibitors
